# Surface Modeling of Workpiece and Tool Trajectory Planning for Spray Painting Robot

**DOI:** 10.1371/journal.pone.0127139

**Published:** 2015-05-18

**Authors:** Yang Tang, Wei Chen

**Affiliations:** 1 School of Science, Jiangsu University, Zhenjiang, China; 2 School of Electronics and Information,Jiangsu University of Science and Technology, Zhenjiang, China; Glasgow University, UNITED KINGDOM

## Abstract

Automated tool trajectory planning for spray-painting robots is still a challenging problem, especially for a large free-form surface. A grid approximation of a free-form surface is adopted in CAD modeling in this paper. A free-form surface model is approximated by a set of flat patches. We describe here an efficient and flexible tool trajectory optimization scheme using T-Bézier curves calculated in a new way from trigonometrical bases. The distance between the spray gun and the free-form surface along the normal vector is varied. Automotive body parts, which are large free-form surfaces, are used to test the scheme. The experimental results show that the trajectory planning algorithm achieves satisfactory performance. This algorithm can also be extended to other applications.

## Introduction

Automated spray painting is an important process in the manufacturing of many products, such as automobiles, furniture, airplanes; etc.Surface modeling is the first step of trajectory optimization for spray painting robot. The shape of workpiece and the tool parameters can strongly influence the quality of painting [1]. In order to achieve the new spraying operation standards, new surfaces modeling of workpiece algorithms for spray painting robot are active research for many years. Surface modeling based on parametric surface in spray painting is presented by Antonio et al [2].For a product with a parametric surface; two basic approaches can be applied. The first one is called the section approach. Spray painting robot trajectories are generated by intersecting the target surface with a series of parallel equidistant section planes. The second is called the offset curve approach that generates a start curve on the target surface, and then constructs the subsequent paths by offsetting the start curve along a family of curves orthogonal to the start curve [3–4]. However, both the two basic approaches are too complicated to model large free-form workpiece surfaces for spray painting [5].

Now, achieving uniform paint thickness for free-form surfaces is still a challenging research topic due to the complex geometry of free-form surfaces[6].And automated trajectory planning has been widely studied.Chen et al. proposed a new optimization algorithm of the path planning for spray painting robot of workpiece surfaces[7]. But the algorithms are too complicated to optimize spray painting trajectory on large free-form surfaces in automobile manufacturing.Chen et al. developed a automatic tool path planning for a free-form surface, and the experimental results illustrate the feasibility and availability of the method[8–9].But their algorithms couldn’t resolve tool trajectory optimization problem.Yu et al. developed an automatic tool path planning for a free-form surface[10–11].Li proposed a new trajectory optimization scheme and a model of paint deposition rate for a free-form surface was established[12]. Li proposed trajectory optimization of spray painting robot for a free-form surface based on adapted genetic algorithm [13].However, due to the process is complex and very time-consuming, their algorithms couldn’t resolve robot trajectory optimization problem in automotive spray painting. The paint thickness function for free-form surfaces is not considered, and the optimal time is not satisfying.

In this paper, a new trajectory optimization scheme for large free-form surfaces in automobile manufacturing is developed. And a free-form surface model is approximated by a set of flat patches. Each patch is treated individually to generate robot trajectories. And a new trajectory optimization scheme by T-Bézier curve is developed. Automotive body parts, which are large free-form surfaces, are used to test the scheme. The results of experiments have shown that the trajectories optimization algorithm achieves satisfactory performance.

## The model of a large free-form surface

The paint deposition rate function on a plane according to the experiment data is considered. And assuming that the shape of spray painting from the gun is a cone and the distribution model of spray is shown in [14].

To obtain time-efficient spray painting robot trajectories and sufficiently utilize the workspace of the robot, a grid approximation of a free-form surface is adopted in CAD modeling. The CAD model of a free-form surface can be formulated as:

$$M=\left\{T_{j}:i=1\ldots M\right\}$$ (1)

Where *T*_*j*_ is the *j*th grid on the free-form surface; *M* is the number of grids.

During spray painting, a free-form surface is only covered by a spray cone at each time instant. The patch forming method is based on minimizing the maximum deviation angle of spray cones. To optimize the paint thickness on a free-form surface, the maximum deviation angle of every spray cone has to be minimized. Assume that there are *N* grids which are covered by a spray cone and the spray cone is projected to a plane. The normal of the plane must be a vector which minimizes the inaxiniuin deviation angle of tile spray cone.

A flat patch is a collection of connected grids, which correspond to a certain area of continuous part surface and satisfy the constraint: the angle between the normal of any grid in the patch and the average normal of the patch is within certain threshold. The average normal of a patch is defined as follows:

$$\vec{n_{a}}=\frac{\sum_{j=1}^{p}s_{j}\vec{n_{j}}}{\sum_{j=1}^{p}s_{j}}/\left\|\frac{\sum_{j=1}^{p}s_{j}\vec{n_{j}}}{\sum_{j=1}^{p}s_{j}}\right\|$$ (2)

Here *s*_*j*_ is the area of grid *Tj*, and $\vec{n_{j}}$ is the normal of *Tj*.

An area-weighted average is employed to calculate the average normal, which reflects the fact that bigger grids have more contribution to the average normal than smaller ones. The average normal is used to indicate the direction of a patch.

The maximum-area-direction of a flat patch is the direction that when the patch is orthographically projected along this direction, the image of the patch has the maximum area. The image area of a patch in orthographic projection with scaling factor *m* = 1 is:

$$S=\sum_{j=1}^{p}s_{j}\left|\vec{n_{j}}\cdot \vec{v_{a}}\right|$$ (3)

Here $\vec{v_{a}}$ is the direction of orthographic projection. To find $\vec{v_{a}}$ such that *S* is maximum, we have:

$$\frac{dS}{d\vec{v_{a}}}=0$$ (4)

The results means the maximum-area-direction is the same direction as the area-weighted “average normal”.

Assume that the material quantity on the flat surface is projected to a free-form surface and the maximum deviation angle of the free-form surface relative to the normal of the flat surface is β_*th*_. The maximum deviation angle is the maximum angle between the normal of the free-form surface and the flat surface. Then the material quantity *q*_*s*_ of each point *s* on the free-from surface can satisfy the following inequality without considering the tool standoff variation:

$${\bar{q}}_{\text{min}}\;\text{cos}(\beta_{th})\leq q_{s}\leq {\bar{q}}_{\text{max}}$$ (5)

Here ${\bar{q}}_{\text{max}}$ 、 ${\bar{q}}_{\text{min}}$ are maximum and minimum material quantity. Then the material quantity of each point *q*_*s*_ in the free-form surface satisfies the constraints:

$$\left|q_{s}-{\bar{q}}_{d}\right|\leq q_{w}$$ (6)

Here ${\bar{q}}_{d}$ 、 *q*_*w*_ are average material quantity and the maximum material thickness deviation.

## Trajectory Optimization based on T-Bézier Curve

Bézier curves are widely used for constructing free-form curves and surfaces[15]. It is well known that the Bézier basis is a basis for the space of degree-*n* algebraic polynomials as:

$$T=span\{1,t,t^{2},\cdots ,t^{n}\}$$ (7)

However, since this basis is rational and polynomial, it would be complicated to use for the tool trajectory of a spray painting robot. This is because each point is associated with six parameters which define the position coordinates and the orientation vector of the spray. In particular, repeated differentiation of Eq 7 produces curves of very high degree[16]. In order to ensure computational efficiency, finding new bases of Bézier model in new spaces seems to be the only way.

In this paper, a new T-Bézier basis is presented in tool trajectory optimization problem of spray painting robot. We first give four initial functions:

$$\begin{array}{l} B_{0,3}(t)={\left(\text{cos}\;t\right)}^{4}\\ B_{1,3}(t)=2{\left(\text{cos}\;t\right)}^{4}{\left(\text{sin}\;t\right)}^{2}\\ B_{2,3}(t)=2{\left(\text{sin}\;t\right)}^{4}{\left(\text{cos}\;t\right)}^{2}\\ B_{3,3}(t)={\left(\text{sin}\;t\right)}^{4} \end{array}$$ (8)

Where $t\in [0,\frac{\pi }{2}].$ For *n*>3,T-Bézier basis functions are defined as:

$$B_{i,n}(t)={\left(\text{cos}\;t\right)}^{2}B_{i,n-1}(t)+{\left(\text{sin}\;t\right)}^{2}B_{i-1,n-1}(t)$$ (9)

Where *B*_*i*,*n*_(*t*) = 0 for *i*>*n* or *i*<0.

With this basis, the curves share most of the properties as those of the Bézier curves in polynomial space.The T-Bézier basis have the properties as follows:

1. Partition of Unity:

$$\sum_{i=0}^{n}B_{i,n}(t)=1$$ (10)

1. Positivity:

$$B_{i,n}(t)\geq 0$$ (11)

So T-Bézier basis is a blending system.

1. Properties at the endpoints:

$$\begin{array}{l} B_{0,n}(0)=B_{n,n}(\frac{\pi }{2})=1,\\ B_{0,n}(\frac{\pi }{2})=B_{n,n}(0)=0,\\ B_{i,n}(0)=B_{i,n}(\frac{\pi }{2})=0,0<i<n \end{array}$$ (12)

1. Linear independence: *B*_0,*n*_(*t*), *B*_1,*n*_(*t*),⋯,*B*_*n*,*n*_(*t*) are linear independent.
2. Symmetry:

$$B_{i,n}(t)=B_{n-i,n}(\frac{\pi }{2}-t)$$ (13)

1. B-basis property: {*B*_0,*n*_(*t*), *B*_1,*n*_(*t*),⋯,*B*_*n*,*n*_(*t*)} is the normalized B-basis of the space *span*{1, cos*t*,⋯,cos*nt*}. By the properties 1) and 2), we have that T- Bézier Basis is a totally positives basis.

A T- Bézier curve *p*(*t*) of order *n*+1 is defined as follows:

$$p(t)=\sum_{i=0}^{n}B_{i,n}(t)V_{i},\;t\in [0,\frac{\pi }{2}]$$ (14)

Where ${\left\{B_{i,n}(t)\right\}}_{i=0}^{n}$ is the T- Bézier basis, *V*_*i*_ is the control point.

The geometric properties at the endpoints of the T-Bézier curves are obvious from those of the T-Bézier basis:

$$p(0)=V_{0},p(\frac{\pi }{2})=V_{n}$$ (15)

Especially for *n* = 3, suppose $V_{0}^{\left[1\right]},V_{1}^{\left[1\right]},V_{2}^{\left[1\right]},V_{3}^{\left[1\right]}$ and $V_{0}^{\left[2\right]},V_{1}^{\left[2\right]},V_{2}^{\left[2\right]},V_{3}^{\left[2\right]}$ are two adjacent sets of T-Bézier control points. The condition of position continuity (*C* ^0^ continuity) is $V_{3}^{\left[1\right]}=V_{0}^{\left[2\right]}$, and the condition of target continuity (*C* ^1^ continuity) is that $V_{2}^{\left[1\right]},V_{3}^{\left[1\right]},V_{0}^{\left[2\right]}$ and $V_{1}^{\left[2\right]}$ are collinear, containing *C* ^0^ continuity.

The entire T-Bézier curve *P*(*t*) must lie inside its control polygon spanned by *V*_*0*_, *V*_*1*_…*V*_*n*_. This property is a consequence of the property of the T-Bézier basis about partition of unity.

The control points of opposite order define the same curve in a different parameterization, just the opposite direction:

$$p(V_{n},V_{n-1},\cdots ,V_{0};t)=p(V_{0},V_{1},\cdots ,V_{n};\frac{\pi }{2}-t)$$ (16)

which can be checked by comparing the coefficients of *V*_0_, *V*_1_…*V*_*n*_. on both sides of the equation. No plane intersects a T-Bézier curve more often than it intersects the corresponding control polygon.

The shape of a T-Bézier curve is independent of the choice of coordinates, i.e. $p(V_{0},V_{1},\cdots ,V_{n};t)=\sum_{i=0}^{n}B_{i,n}(t)V_{i}$ satisfies the following two equations:

$$\begin{array}{c} p(V_{0}+r,V_{1}+r,\cdots ,V_{n}+r;t)=p(V_{0},V_{1},\cdots ,V_{n};t)+r\\ p(V_{0}{}^{*}T,V_{1}{}^{*}T,\cdots ,V_{n}{}^{*}T;t)=p(V_{0},V_{1},\cdots ,V_{n};t){}^{*}T \end{array}$$ (17)

Here *r* is an arbitrary vector, and *T* is an arbitrary (*n*+1)*(*n*+1) matrix. If the control polygon is convex, then the corresponding T-Bézier curve is also convex.

## Simulation

**Fig 1:**
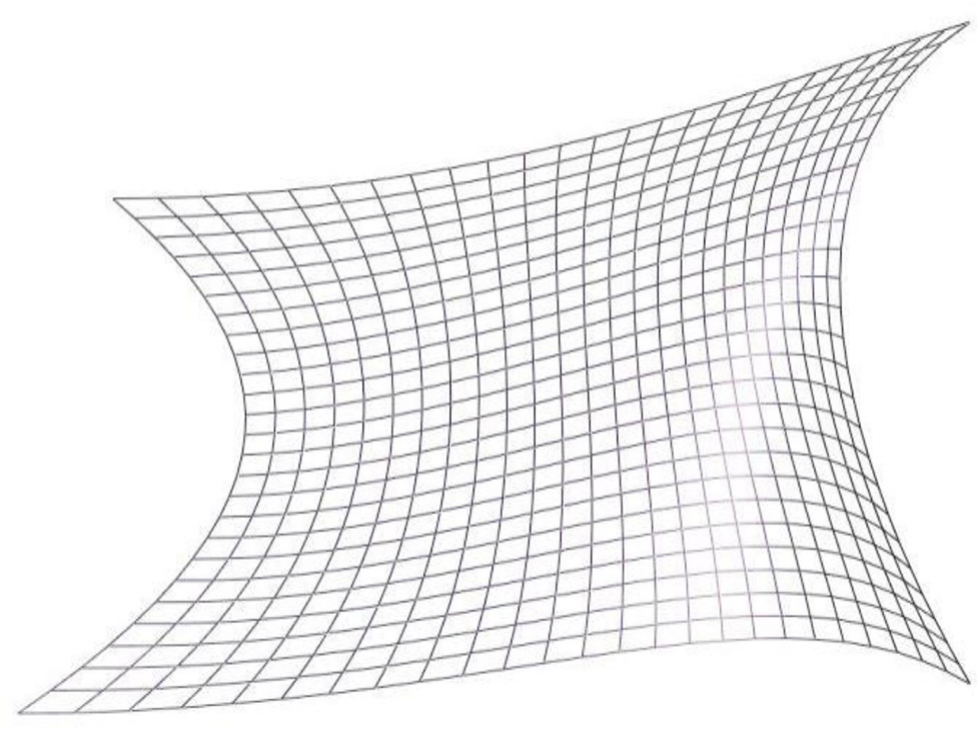
The grids approximation of a free-form surface.

**Fig 2:**
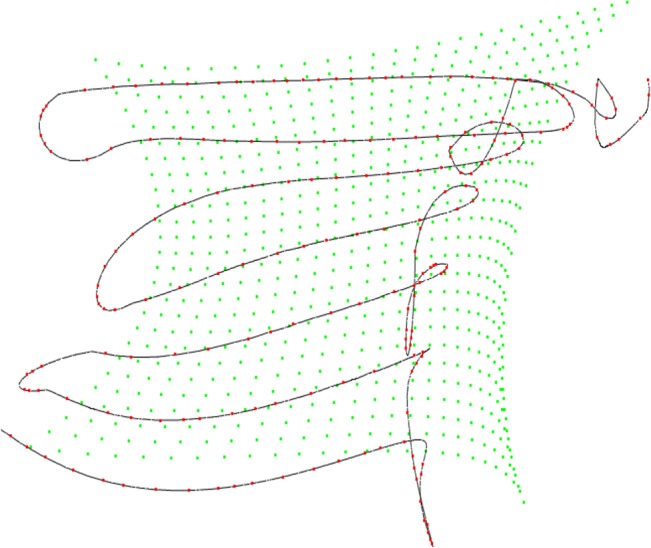
The optimization trajectory on the free-form surface.

The algorithms are implemented in C++, and a free-form surface, shown in Fig 1, is used to test the trajectory optimization algorithm. The control points on the free-form surface are *i* = 212.The tool trajectory is formed through offsetting the distance between spray tool and the free-form surface along the normal vectors. Then the optimization trajectory is generated using T-Bézier curve. The generated tool trajectory is shown in Fig 2. Assuming that the shape of spray painting from the tool is a cone and the distribution model of spray is shown in [14]. Suppose the required average material thickness is *q*_*s*_ = 50μm, and the max material thickness deviation is *q*_*w*_ = 10μm.The spray radius is *R* = 60mm. The material deposition rate is:

$$f(r)=\frac{1}{15}(R^{2}-r^{2})\mu m/s$$ (18)

The results for optimal tool trajectories planning are summarized in Table 1.

**Table 1:** The results for optimal trajectories planning of the simulation.

	Material thickness of the 212 control points on the free-form surface
Average(μm)	49.7
Maximum(μm)	56.9
Minimum(μm)	48.1
Process time(s)	86

## Experimental verification

Automotive body parts from a car company are tested. And the spraying parameter settings in experiment are the same to the simulation.

**Fig 3:**
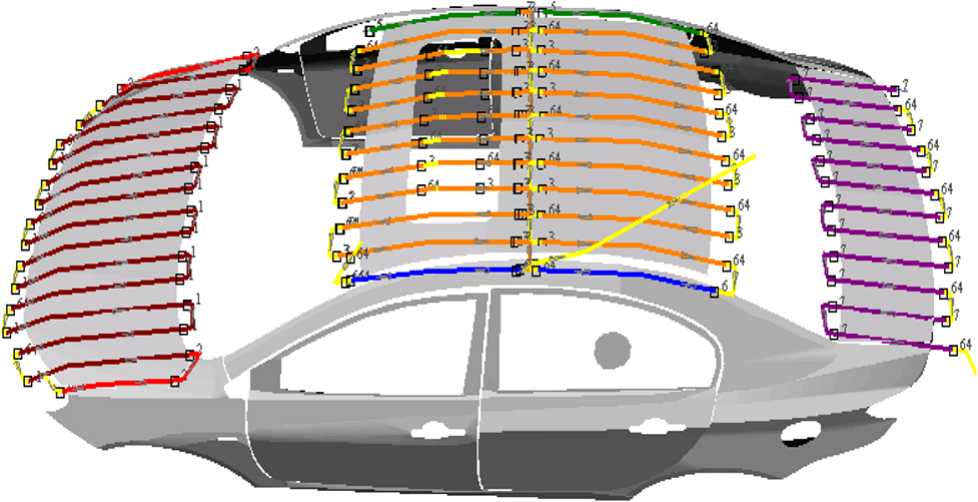
The optimal tool trajectories on the car roof.

**Fig 4:**
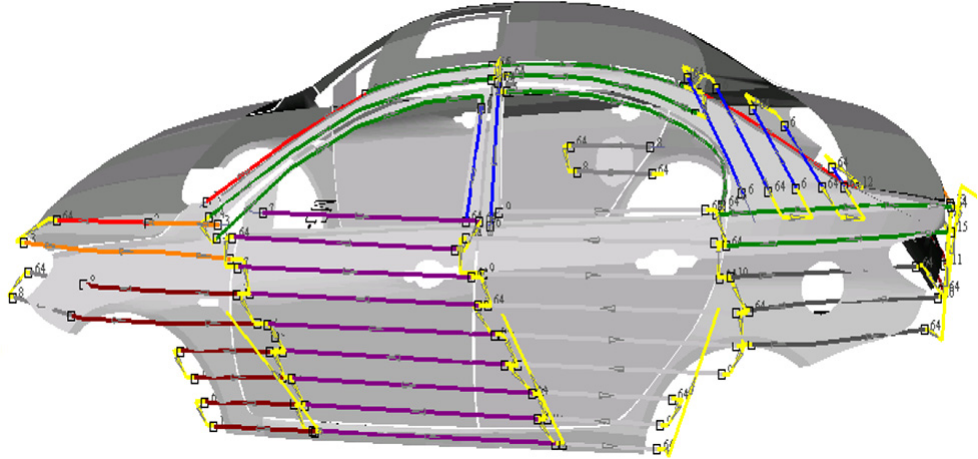
The optimal tool trajectories on the car left body.

**Fig 5:**
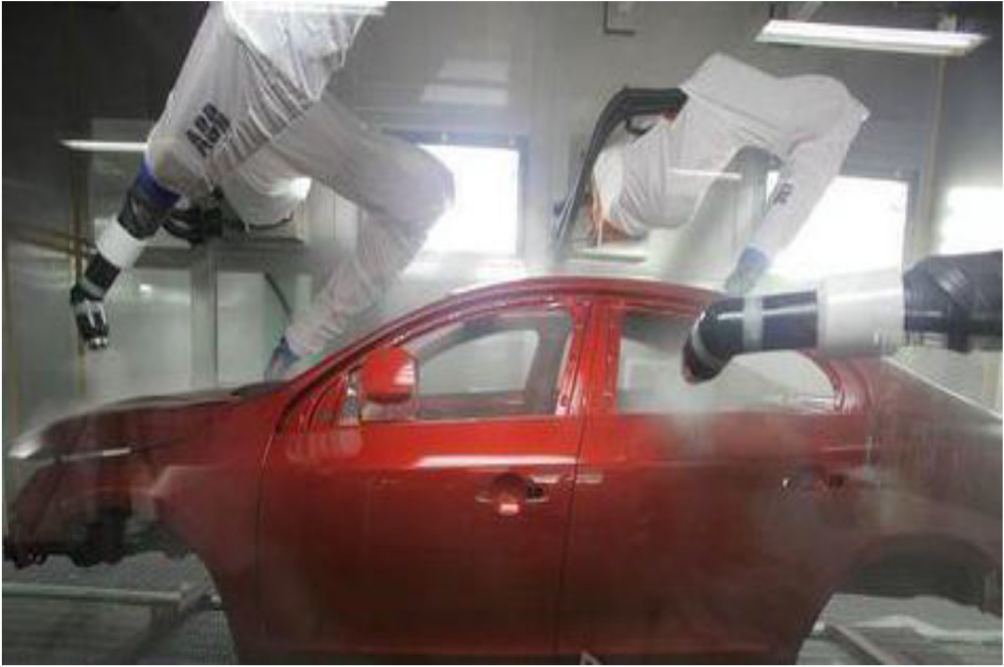
Robotic spray painting experiment.

The first step is surfaces modeling. And a free-form surface model is approximated by a set of flat patches. The second step is trajectory optimization. We first determined the optimal movement patterns and sweeping directions, according to which the final trajectories are generated. Then the optimization trajectory is generated using the T-Bézier curves by the control points. The optimal tool trajectories on the car roof is shown in Fig 3.The optimal tool trajectories on the car left body is shown in Fig 4 and Fig 5 shows the robotic spray painting experiment.

**Fig 6:**
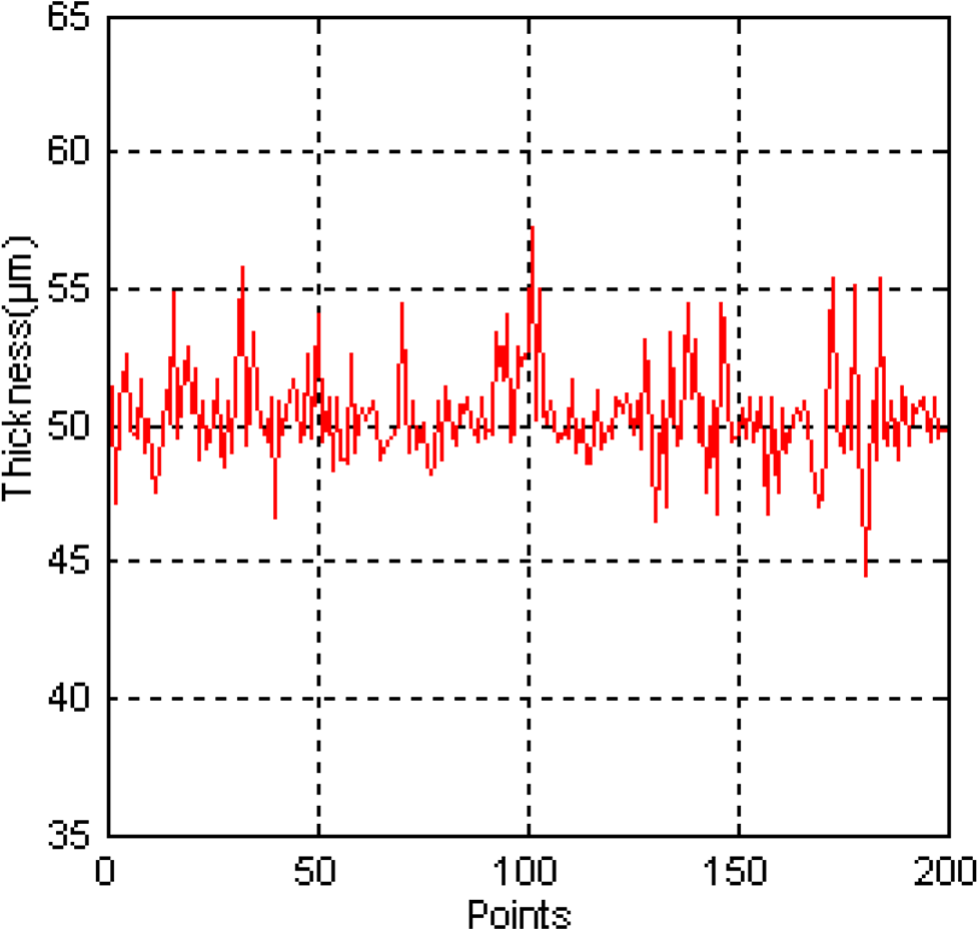
Material thickness of random chosen points on the car body.

The thickness of the coating at randomly chosen points on the car body is measured using the Elcometer 456 automobile coating thickness gauge which resolution ratio is 1μm. Fig 6 shows the results for material thickness of 200 chosen points along the direction of spray painting trajectory on the car body. The results for optimal tool path planning are summarized in Table 2.

**Table 2:** The results for optimal trajectories planning of the experiment.

	Material thickness
Average(μm)	49.5
Maximum(μm)	57.2
Minimum(μm)	44.5
Process time(s)	493

## Conclusion

A grid approximation of a free-form surface is adopted in CAD modeling. And a free-form surface model is approximated by a set of flat patches. Each patch is treated individually to generate robot trajectories. A new trajectory optimization scheme based on T- Bézier curve is developed. Automotive body parts, which are free-form surfaces, are used to test the scheme. And the results demonstrate the advantages of the optimal trajectories planning algorithm. This algorithm can also be extended to other applications such as optimal tool path for free-form surface of cleaning robot or grinding robot, etc.
